# Mr. Ralph Arthur John Baily

**Published:** 1991-03

**Authors:** 


### Obituaries

### Ralph Arthur John Baily 1919–1990

John Baily, the well known, and well loved Orthopaedic Surgeon who had worked in Weston-super-Mare, as a Consultant for 33 years, died on 8.10.90 after a brief illness, when the problems with his heart, which had started some 20 years previously, finally overcame his indomitable spirit.
Ralph Arthur John Baily was born on 29.6.19. His father was at sea in the early part of his life and, in consequence, he lived with his grandparents in Glasgow, and the first school he attended was Glasgow Academy. Perhaps this Scottish grounding contributed to his subsequent thoroughness and ability to work hard.

Thereafter he went to Cranleigh School, and on to Clare College, Cambridge. He had decided to do Medicine before going on to Cambridge and was accepted for Guy's Hospital Medical School after doing the first part of his MB exams. He qualified M.B. B.Chir. Cambridge, in 1943, and after a couple of House jobs joined the Navy, where, as a Surgeon Lieutenant, he was the Medical Officer responsible for the health of a flotilla of mine sweepers in home waters.

When he was demobilized he joined the Royal Naval Reserve, and continued to have an interest in the Royal Naval Reserve until he retired from it in 1960 with the Reserve Decoration.

His career as an Orthopaedic Surgeon started when he joined the Ipswich Hospital, where he first came in touch with Orthopaedics and had a lot of "hands on" experience during his time in Ipswich. His interest in Orthopaedics was firmly established.

He then came to Bristol and worked as a Registrar at Winford Orthopaedic Hospital. He moved from there to Southampton, where he was a Senior Registrar in Orthopaedics, and in 1953 obtained the fellowship of the English College. Shortly afterwards he was appointed Senior Registrar at the Bristol Royal Infirmary, where he spent three years working both at the Teaching Hospital, and at Winford Orthopaedic Hospital, and was made a Tutor in Orthopaedic Surgery in the University of Bristol.

At the end of this period in 1957 he was appointed Consultant Orthopaedic Surgeon at Weston General Hospital, and Winford Orthopaedic Hospital. He worked there until he retired in 1983.

Working at Weston-super-Mare, at first single handed, and even after the appointment of a colleague to share the workload, was always arduous, often with few junior staff and a one in two on call commitment. He never seemed dismayed and loyally carried out his full share of the work.

During this time he established a reputation for not only being a delightful colleague, but also an extremely effective surgeon. He never became flustered. He rarely had reason to become flustered, as his technique was such that operations always went smoothly. So much so that his Assistants often did not realize the problems involved in the operation until they came to do it themselves.

He developed a particular interest in knee surgery, and with other consultants in the area developed the Bristol Knee Group. As he had already seen the value of computer records and had become very interested in them, early on in the history of the knee group all the records were placed on his computer so that they were remarkably easy of access, and well organised for those years.

He established a large private practice, particularly in hip and knee replacement surgery, and was undoubtedly for many years "the Orthopaedic Surgeons Orthopaedic Surgeon". So that he dealt with many colleagues and their wives. To do this he had to travel to Bristol, twenty miles each way, for every visit to his patients, which must have added to the stresses and strains involved.

His hobbies were photography and he was very skilled at this. He made his own slides, printed his own colour prints and had his own extensive dark room at home.

He was also a very keen sailor and the first thing he did when he retired from the NHS in 1983 was to arrange a crew and take his 30ft. Catamaran from Plymouth to Athens. Many stories have been told about this voyage, some of which no doubt are apocryphal. He was also interested in wine and was a member of one of the local Wine Clubs.

With other members of the South West Orthopaedic Club, he frequently joined the annual meetings of the Orthopaedic Club of the West of France, sailing there on one occasion in his own boat. It was interesting that although he had quite a marked stutter all his life, when he got up to give a paper whether it be in English or French, this stutter seemed to disappear!

One of his other interests was World Orthopaedic Concern, and he was considerably engaged in the organisation of the service which W.O.C. provided in Bulawayo.

From all this list of activities one would hardly believe that 21 years ago when he was sailing out of the harbour at Brixham he suffered a severe coronary and was detained in Torbay Hospital for something like three weeks. He never let this hold him back. He worked extraordinarily hard both in the NHS and in private. He still indulged in all his hobbies, including sailing.

His final illness started when he and his wife, Bette, were on a Wine Tasting Holiday in Northern Italy and Southern Yugoslavia and he began to develop severe angina. In spite of treatment in the Coronary Care Unit at the Bristol Royal Infirmary when he managed to get back from Yugoslavia, he died within a short while of the onset of these symptoms.

As one of his colleagues over all these years, one can only say that John was an outstanding Orthopaedic Surgeon, an outstanding person, and someone who stimulated love and affection from all those who knew him. Our sympathy goes out to his widow, Betty, and his children, Gillian, David, Patricia, and Guy (Guy being a doctor).

M.P. McC.

